# The analgesic efficiency of pregabalin for the treatment of postoperative pain in total hip arthroplasty: A randomized controlled study protocol

**DOI:** 10.1097/MD.0000000000021071

**Published:** 2020-07-02

**Authors:** Yuangui Zhang, Xiaoqian Wang, Guimin Dong

**Affiliations:** Department of Anesthesiology, Weifang People's Hospital, Shandong 261000, China.

**Keywords:** pain control, pregabalin, randomized controlled trial, study protocol, Total hip arthroplasty

## Abstract

**Background:**

Only few studies have yet investigated whether perioperative administration of pregabalin can reduce the incidence of postoperative chronic neuropathic pain after total hip arthroplasty (THA). This prospective, randomized study compared placebo with pregabalin in the hope that a lower pregabalin dose would improve analgesia without increasing side-effects after THA.

**Methods:**

This study was a prospective randomized blinded study, with a parallel design and an allocation ratio of 1:1 for the treatment groups. The study was approved by the Institutional Review Board in Weifang People's Hospital and written informed consent was obtained from all subjects before enrolment. A total of 120 patients who meet inclusion criteria are randomized to either pregabalin or placebo group. The primary objective of the study was visual analog scale score. As secondary outcomes, opioid consumption measurement, Harris Hip Score, hip range of motion, patient satisfaction, and complications were made at different time points throughout the study for comparison.

**Results:**

The null hypothesis of this study was that pregabalin would reduce pain after THA.

**Trial registration:**

This study protocol was registered in Research Registry (researchregistry5669).

## 1 Introduction

Total hip arthroplasty (THA) is one of the most effective orthopedic procedures to relieve pain and improve function in patients with symptomatic hip arthritis.^[1]^ It is projected that the number of THAs per year is approximately 400,000, and the amount has been increasing by 25% to 30% per year in China.^[2]^ THA is known to be a painful orthopedic procedure and moderate to severe pain is common, especially immediately postoperatively and during active motion. Poorly controlled pain can result in poor function with physical therapy, prolonged hospitalization, and reduced patient satisfaction.^[3]^ Therefore, adequate analgesia following THA continues to be a topic of interest.

Despite advances in surgical technology and perioperative anesthetic management, the incidence of chronic neuropathic pain after THA surgery has not decreased.^[4–6]^ Neuropathic pain is a distressing condition that is characterized by allodynia, hyperalgesia, edema, and skin color changes of the limb. Treatment of neuropathic pain is often both challenging and prolonged, with substantially diminished quality of life. Gabapentin and the related more potent compound pregabalin have been shown to be beneficial in the treatment of neuropathic pain.^[7–9]^ Because of the chronic and distressing nature of neuropathic pain, as well as the difficulty in treatment and resolution, preventing development of this syndrome is highly advantageous.

Pregabalin is an analog of gamma aminobutyric acid. Pregabalin decreases the synaptic release of multiple neurotransmitters (glutamate, noradrenaline, serotonin, dopamine, and Substance P) by binding to the α2δ-subunits of the presynaptic voltage-dependent calcium channels.^[10,11]^ As a result, the drug inhibits neuronal excitability, especially in the central nervous system.^[11–13]^ Pregabalin has been shown in multiple studies to be an effective analgesic agent in both peripheral and central neuropathic pain,^[14–16]^ and it is also effective in reducing post-THA pain.^[4–6]^

However, only 3 clinical studies have yet investigated whether perioperative administration of pregabalin can reduce the incidence of postoperative chronic neuropathic pain after THA.^[4–6]^ This prospective, randomized study compared placebo with pregabalin in the hope that a lower pregabalin dose would improve analgesia without increasing side-effects after THA. The null hypothesis of this study was that pregabalin would reduce pain after THA.

## 2 Materials and methods

This study was a prospective randomized blinded study, with a parallel design and an allocation ratio of 1:1 for the treatment groups. The study was approved by the Institutional Review Board in Weifang People's Hospital (WF2020010058) and was registered in the Research Registry (researchregistry5669). Written informed consent was obtained from all subjects before enrolment. This study was performed and reported in accordance with the principles of the Declaration of Helsinki (2000).

### 2.1 Patients

Eligible patients were 18- to 80-year-old Chinese speakers judged able to follow the protocol, planned to undergo primary THA and regional anesthesia and to be discharged home or to a participating rehabilitation center. Exclusion criteria included planned general anesthesia, allergy, or intolerance to one of the study medications, American Society of Anesthesiologists physical status of IV, hepatic failure, renal failure, difficult-to-manage diabetes mellitus (including insulin dependence), chronic gabapentin or pregabalin use (regular use for longer than 3 months), and chronic opioid use (regular use for longer than 3 months).

### 2.2 Randomization

**Figure 1:**
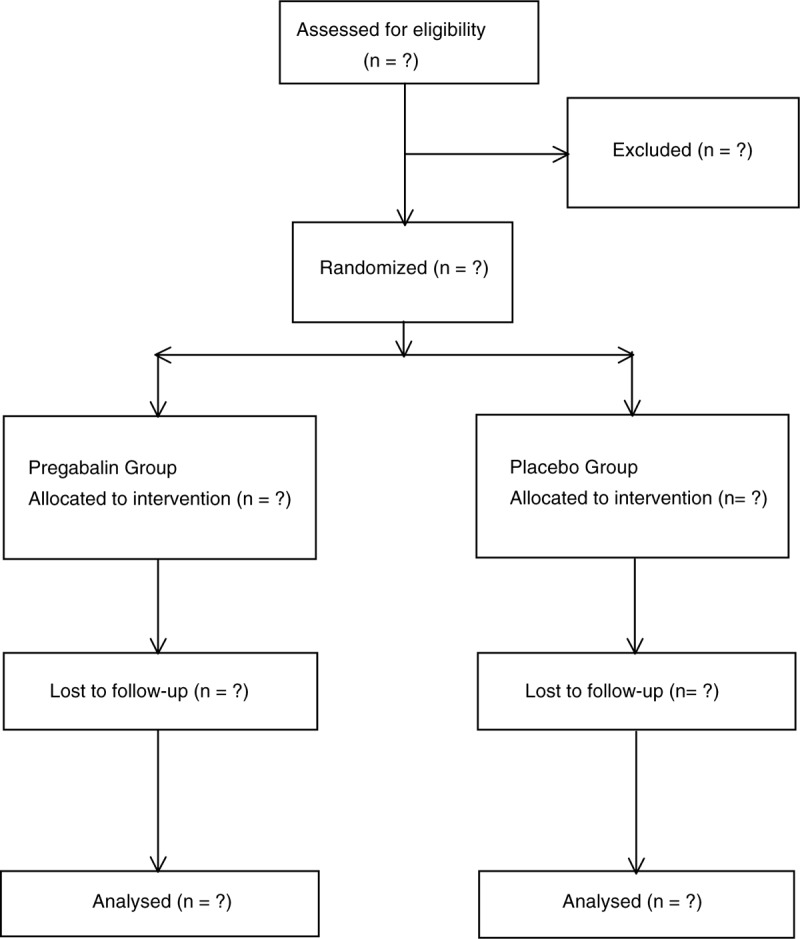
Flow of patients through the trial.

An equal number of envelopes for each treatment group were prepared using a computerized random number generator by a study assistant who did not take part in any subsequent part of the study, and was not in contact with the rest of the study team throughout the entire study duration. He prepared 120 identical sequentially numbered, opaque, sealed and stapled envelopes; 60 envelopes contained instructions for mixing solutions for pregabalin group, and the other 60 for placebo group. The envelopes were kept in a file with the principal investigator (Fig. 1).

### 2.3 Surgical techniques

Indications for THA were severe hip pain and/or considerable difficulty in walking and performing daily activities with significant hip osteoarthritic changes, complete hip joint dislocation, or an ankylosed hip joint. All THAs in this study were carried out by either the senior author, or by fellows under his direct supervision. A tourniquet was used in all cases, general anesthesia was administered to each patient before incision, and the operative hip was prepared and draped in a conventional sterile fashion.

### 2.4 Intervention

Patients were randomly assigned to receive either the study medication or placebo. There was no dose administered on the days before surgery. Patients randomized to the experimental arm of the study received pregabalin 300 mg orally, 1 to 2 hours before surgery, 150 mg twice daily for the first 10 postoperative days, 75 mg twice daily on days 11 and 12, and 50 mg twice daily on days 13 and 14. The placebo group received matching placebo tablets bi-daily for the duration of the treatment period. Our institutional standard regimen for pain control consisted of either intravenous morphine preservative-free 2 mg every 2 hours as needed or hydromorphone injection 0.5 mg every 2 hours as needed for severe pain and oxycodone (immediate release) tablet 5 mg every 6 hours as needed for moderate pain. Subjects received intravenous and/oral opioid medication as pain rescue during hospitalization and subsequently, opioid consumption during hospitalization was collected and converted to oral morphine units for data analysis purposes.

### 2.5 Outcome measures

The primary objective of the study was to evaluate the efficacy of pregabalin compared to placebo in the reduction of pain in THA patients. The severity of pain was assessed by using the visual analog scale (VAS) for pain intensity; it consisted of a 100 mm long line with 2 descriptors at each end representing pain intensities (no pain and extreme pain). Subjects were asked to mark their pain intensity somewhere on the line, then the VAS score was assessed by measuring the distance from the “no pain” end to the mark placed by the subject. VAS pain assessments were performed at baseline, on day 3, day 9 ± 3, day 25 ± 7, day 90 ± 6, and day 180 ± 12.

As secondary outcomes, opioid consumption measurement, Harris Hip Score, hip range of motion, patient satisfaction, and complications were made at different time points throughout the study for comparison. Opioid consumption (oral morphine per day) before first dose of investigational product until hospital discharge or day 3 (whichever occurs first) was recorded. Harris Hip Score was originally developed to evaluate the treatment of posttraumatic arthritis, but is now widely used for any osteoarthritis of the hip, and has been found to be responsive for these patients. The score has a maximum of 100 points (best possible function), covering pain (1 item, 0–44 points), function and activities (7 items, 0–47 points), and range of motion and absence of deformity (3 items, 0–9 points). Range of motion, Harris Hip Score and patient satisfaction were obtained both before and after arthroplasty by a dedicated physiotherapist specialist.

### 2.6 Statistical analysis

The sample size was determined for the primary endpoint and was calculated using PASS 2011 software (NCSS, LLC, Kaysville, UT). According to the results of our previous study, the postoperative VAS score was 2.16 in the control group. We anticipated a difference of 0.72 in the VAS score. With a power of 0.90 and significance level of 0.05, the required sample size was calculated as 50 in each arm. Considering possible exclusion, we decided to include 60 patients in each group. Statistical analysis was performed by an independent expert, not involved in the study protocol. Median and mean (range) values were presented. The unpaired *t* test was used for the normally distributed numerical data. A nonparametric analog was used where appropriate. The χ^2^ test was used to compare categorical variables. A *P* value <.05 was considered statistically significant.

## 3 Discussion

Pregabalin is an α 2 δ ligand that modulates the activity of voltage gated calcium channels. In the US, pregabalin is indicated for the treatment of neuropathic pain associated with diabetic peripheral neuropathy, with spinal cord injury, postherpetic neuralgia, fibromyalgia, and as an adjunctive therapy for adult patients with partial onset seizures.^[17]^ Some studies show that pregabalin given before surgery can reduce dental pain after molar extraction,^[18]^ reduce postoperative morphine requirements after THA,^[4–6]^ and attenuate postoperative pain after laparoscopic cholecystectomy^[19]^; however, other studies show no beneficial effect of pregabalin on acute postoperative pain when administered preoperatively for minor gynecological surgery.^[20]^

This prospective, randomized study compared placebo with pregabalin in the hope that a lower pregabalin dose would improve analgesia without increasing side-effects after THA. The strength of this study was its prospective and randomized design. Three studies have recently been published evaluating the effects of pregabalin in THA. The present study continues to provide ongoing data showing the differences in the clinical results between the 2 groups.

## Author contributions

**Conceptualization:** Yuangui Zhang.

**Data curation:** Xiaoqian Wang.

**Formal analysis:** Yuangui Zhang, Xiaoqian Wang.

**Funding acquisition:** Guimin Dong.

**Investigation:** Yuangui Zhang, Xiaoqian Wang.

**Methodology:** Guimin Dong.

**Resources:** Guimin Dong.

**Software:** Xiaoqian Wang.

**Supervision:** Guimin Dong.

**Validation:** Yuangui Zhang.

**Visualization:** Yuangui Zhang.

**Writing – original draft:** Yuangui Zhang, Xiaoqian Wang.

**Writing – review & editing:** Guimin Dong.
